# Management of Direct Oral Anticoagulants in Acute Type A Aortic Dissection

**DOI:** 10.1055/a-2542-4290

**Published:** 2025-05-08

**Authors:** Robert Semco, Thais Faggion Vinholo, Jake Awtry, Asishana Osho, Kim de la Cruz, Ashraf A. Sabe

**Affiliations:** 1 Harvard Medical School, Boston, Massachusetts; 2 Division of Cardiac Surgery, Department of Surgery, Brigham and Women's Hospital, Boston, Massachusetts; 3 Division of Cardiac Surgery, Department of Surgery, Massachusetts General Hospital, Boston, Massachusetts

**Keywords:** direct-acting oral anticoagulants, aortic dissection, anticoagulation reversal

## Abstract

**Background** Direct oral anticoagulants (DOACs) are a commonly used class of anti-coagulants that may complicate surgical management of acute Type A aortic dissection (ATAAD).

**Methods** Surgical management and clinical courses were described for patients who presented to our institution with ATAAD while taking DOACs, after FDA approval of the two currently available reversal agents. A thorough literature review was completed for cases of administration of DOAC reversal agents in ATAAD.

**Results** The only patient treated with andexanet-alfa had heparin insensitivity while on cardiopulmonary bypass. Four other patients were successfully managed with a combination of surgical delay and factor repletion.

**Conclusion** This case series demonstrates that preoperative management of DOACs in patients with ATAAD may employ factor repletion with success. Literature review demonstrated a safety signal for heparin insensitivity or pump thrombosis when andexanet-alfa was administered before or while on cardiopulmonary bypass or extracorporeal membrane oxygenation. Our institutional clinical practice guidelines recommend against administration of andexanet-alfa within 4 to 6 hours before heparinization for surgery in ATAAD but recommend considering andexanet-alfa administration when there is life-threatening bleeding after heparin reversal that is thought to be due to Xa-inhibition with laboratory evidence of elevated anti-Xa activity.

## Introduction

Direct oral anticoagulants (DOACs) are a class of medications that are commonly encountered in cardiac surgery and include the factor Xa-inhibitors apixaban, rivaroxaban, and edoxaban and thrombin inhibitor dabigatran. However, despite their prevalence, evidence on how to proceed with emergent cardiac surgical procedures in the setting of recent DOAC administration is scarce. 1 2 While it is standard practice to await clearance of the DOAC when surgery can be delayed, proper management is less clear when cardiac surgery is emergently indicated. With regard to acute Type A aortic dissection (ATAAD), a clinical condition distinguished from other emergent cardiac surgical conditions by the high risk of preoperative and postoperative bleeding and thrombosis, evidence for how to manage DOACs is limited to case reports and small case series. 3 4 5 6 7 8 9 10 11 12 Nonetheless, it is known that DOACs carry increased risk for uncontrolled bleeding and prolonged operative time in ATAAD compared with vitamin K antagonists. 13 Furthermore, DOACs are associated with higher mortality after surgery for ATAAD compared with no anticoagulation at all. 13 14

There are two FDA-approved reversal agents for DOACs. Andexanet-alfa is a recombinant factor Xa that competitively reverses Xa-inhibitors (approved for apixaban and rivaroxaban). 15 16 Idarucizumab is a monoclonal antibody that reverses the effects of the thrombin inhibitor dabigatran. 17 In 2020, the European Medicines Agency released a communication warning against use of preoperative andexanet-alfa in cardiac surgery due to risk of heparin insensitivity. 18 However, in 2021, the Society for Thoracic Surgeons (STS) still recommended use of andexanet-alfa or idarucizumab in patients on DOACs before emergent cardiac surgery. 19 A recent review of case reports and series, describing 9 patients on Xa-inhibitors and 84 patients on dabigatran who were undergoing emergent cardiac surgical procedures, found that administration of andexanet-alfa before cardiopulmonary bypass (CPB) was associated with heparin insensitivity, sometimes leading to thrombosis, but that idarucizumab was safe for use at any phase of surgery. 1 They recommended use of prothrombin complex concentrate (4F-PCC) in patients on Xa-inhibitors and the use of idarucizumab in patients on thrombin inhibitors undergoing emergent cardiac surgery. 1 Another recent review reported similar findings, adding that hemoadsorption of DOACs and P2Y12 inhibitors may also be safe and effective to reverse anticoagulation and antiplatelet effects. 2 Nonetheless, evidence supporting or discouraging use of andexanet-alfa or idarucizumab for reversal of DOACs before or after emergent surgery for ATAAD remains limited. Therefore, we conducted a retrospective case series of patients with recent DOAC ingestion undergoing emergent aortic surgery for ATAAD.

## Materials and Methods

All patients who underwent aortic repair or replacement for ATAAD from October 2015 through December 2021 were screened in this retrospective chart review. Inclusion criteria for the case series included (1) presentation for ATAAD, defined as symptom onset within the last 2 weeks, (2) underwent aortic repair or replacement, (3) was prescribed outpatient DOACs at the time of dissection and reported a dose within the last 3 days or had laboratory evidence of elevated anti-Xa activity. Of 136 patients who were screened, 6 patients met inclusion criteria. However, one patient did not provide consent, resulting in a series comprising of five patients. Consent to publish clinical information was obtained from all other patients or from their proxies, with the exception of one patient who was lost to follow-up. Institutional review board approval was not required to publish this case series. Their presentations and clinical courses are described below and in Table 1 .

**Table 1:** Clinical information, reversal agents, and outcomes for patients presenting with acute Type A aortic dissection on DOACs

Patient	Age	Sex	Time to presentation	Procedure	preoperative anticoagulant	Last dose	Reversal agents given	Other factors	Operative mortality	In-hospital mortality	Postoperative bleeding	Thrombosis or heparin insensitivity
1	82	M	5 d	Aortic root repair, aortic valve resuspension, ascending aortic replacement	Apixaban 5 mg twice a day	Same day as presentation	None	7,864u 4F-PCC, 1u FFP, aminocaproic acid	No	No	No	No
2	73	M	24 h	Ascending aortic aneurysm repair and total arch replacement with elephant trunk	Apixaban 2.5 mg twice a day	Not stated, but apixaban calibrated anti-Xa level of 40 ng/mL	None	4,352 4F-PCC, 14,974 mg fibrinogen, 7u FFP, aminocaproic acid	No	No	No	No
3	79	F	1 day	Bio-bentall with hemiarch replacement	Rivaroxaban 20 mg	24 h prior to presentation	Surgery delayed 2 d	2,000 u 4F-PCC, 2u FFP, 6,853 mg fibrinogen, 1 mg recombinant factor VIIa	No	No	No	No
4	78	F	<12 h	Ascending aorta and aortic arch replacement with elephant trunk	Apixaban 5 mg twice a day	10 h prior, anti xa 1.94	None	3,967 mg PCC, 250 u aPCC, 3u FFP, 2,282 mg fibrinogen	No	No	No	No
5	46	M	2 d	Aortic root repair	Apixaban 5 mg twice a day	Same day as presentation	1,760 mg andexanet-alfa pre-CPB	None	No	No	No	Heparin insensitivity

## Results

### Case 1

Patient one was an 82-year-old man with a history of hypertension, prior stroke without atrial fibrillation on apixaban 5 mg twice a day, abdominal aortic aneurysm status after endovascular aneurysm repair, and peripheral artery disease who presented with 5 days of neck and upper chest pain. He last took apixaban the morning of his presentation. He presented to our institution after an outpatient computed tomography (CT) demonstrated a crescentic intramural hematoma of the ascending aorta extending from the sinotubular junction (STJ) to the mid transverse arch as well as a small pericardial effusion with active extravasation. His fondaparinux-calibrated anti-Xa level on presentation was 1.89 mg/L (discordant assay, reference: prophylactic dosing 0.39–0.5 mg/L).

He was taken emergently to surgery and was given 2,949 units 4F-PCC immediately prior to incision. He underwent successful aortic root repair, aortic valve resuspension, and replacement of the ascending aorta. After weaning from CPB, the patient was given another 4,915 units of 4F-PCC, 1 unit of fresh-frozen plasma (FFP), and 1 unit of platelets.

His postoperative course was complicated by a pleural effusion requiring thoracentesis on postoperative day (POD) 14 and pneumonia requiring antibiotics. He was discharged to a rehabilitation facility on POD 18 but passed away 15 days later from unspecified causes.

### Case 2

Patient two was a 73-year-old man with a history of hypertension, unprovoked pulmonary embolism now on apixaban 2.5 mg twice a day, and a known thoracoabdominal aortic aneurysm, who presented 24 hours after acute onset of substernal chest pain with radiation to the back. The time of the last dose of apixaban was unknown, but his apixaban-calibrated anti-Xa level was 40 ng/mL (reference: peak 59–321ng/mL, trough 22–230ng/mL). Computed tomography angiography (CTA) demonstrated a ATAAD extending from the STJ to the level of the infrarenal abdominal aorta, also involving the right brachiocephalic artery, the right subclavian artery, and the right common carotid artery.

He was taken emergently to the operating room (OR) where he underwent aortic valve resuspension, Sinus of Valsalva repair, ascending aorta repair and total arch replacement with an elephant trunk graft extending into the distal aorta. Notably, there was bleeding from the descending aorta beyond the distal suture line, which was controlled with pledgeted sutures. Upon decannulation, there was a sudden increase in bleeding from the aortic root at the noncoronary sinus of Valsalva, so CPB was reinitiated and the area was repaired. In total, he received 4,352 units of 4F-PCC, 14,974 mg of fibrinogen, 3 units of packed red blood cells (pRBCs), 7 units of FFP, and 4 units of platelets.

His postoperative course was complicated by concern for pulmonary embolism requiring a titratable heparin drip, a right basal ganglia stroke secondary to right common carotid dissection, tachycardic–bradycardic syndrome with atrial fibrillation and atrial flutter, delirium, and severe *Clostridium difficile* colitis. He also required an additional unit of pRBCs on POD 3. He was medically ready for discharge on POD 35 and was discharged to a rehabilitation facility.

### Case 3

Patient three was a 79-year-old woman with a history of hypertension, atrial fibrillation on rivaroxaban 20 mg, and pericarditis status after pericardial window who was transferred from an outside hospital (OSH) with 1 day of chest pain and a CTA notable for an intramural hematoma involving the aortic root and ascending aorta. Her last dose of rivaroxaban was taken 24 hours prior to presentation and her fondaparinux-calibrated anti-Xa activity level on admission was 0.76 mg/L (discordant assay, reference: prophylactic dosing 0.39–0.5 mg/L). Given her age and hemodynamic stability, combined with the surgical team's expectation for extensive surgery and unfamiliarity with idarucizumab, surgery was delayed and she was admitted to the intensive care unit to allow rivaroxaban washout.

Two days later, she underwent a bio-Bentall procedure with right coronary artery reconstruction and hemiarch replacement. After rewarming was complete and the patient had been weaned from CPB, significant coagulopathy was encountered but was successfully managed with topical hemostatic agents, 2,000 units 4F-PCC, and 6,853 mg fibrinogen. Intraoperatively, she received 2 units of pRBCs, 2 units FFP, 3 units of platelets, and 350 mL of cell-saver RBCs. Immediately postoperatively, she continued to have sanguineous chest tube output and was given 1 mg recombinant Factor VIIa. Over the remainder of her admission, she received an additional 2 units of pRBCs.

Her postoperative course was otherwise notable for the development of atrial fibrillation on POD 3, pneumonia complicated by respiratory failure requiring reintubation on POD 4, thoracentesis of bilateral loculated pleural effusions on POD 10, and gradual improvement of respiratory status with extubation on POD 11. Following extubation, she was found to have multiple early and late subacute cerebral subacute infarcts, most likely of cardioembolic etiology. She was started on warfarin for stroke prevention on POD 20 and was discharged to a rehabilitation facility on POD 22. Unfortunately, she passed away 7 days later, although the details of her death are not available to our institution.

### Case 4

Patient 4 was a 78-year-old woman with a history of hypertension and paroxysmal atrial fibrillation on apixaban 5 mg twice a day who was transferred from an OSH with sharp chest pain that radiated to her back. She was found to have an ATAAD that extended from the aortic root to the left external iliac artery, with the celiac and left renal arteries arising from the false lumen. She reported last taking apixaban approximately 8 hours prior, and her heparin-calibrated anti-Xa activity level on admission was 1.94 IU/mL (discordant assay, therapeutic range: 0.3–0.7 IU/mL).

She was taken emergently to the OR where she underwent repair of the sinus of Valsalva, resuspension of the aortic valve, total arch replacement, and elephant trunk positioned in the descending aorta with reimplantation of the brachiocephalic and left carotid arteries, sacrificing the left subclavian artery. After decannulating from CPB, her anti-Xa activity was 0.68, so the decision was made to not administer andexanet-alfa. Intraoperatively, she received a total of 1,050 mL cell-saver RBCs, 4 units of pRBC, 4 units of platelets, 3 units FFP, 2,282 mg fibrinogen, 3,967 units 4F-PCC, and 250 units activated PCC (aPCC).

On POD 1, due to increased chest tube output she was given 1 unit of platelets. Her postoperative course was otherwise notable for paroxysmal atrial fibrillation and atypical flutter managed with amiodarone, delirium, and a urinary tract infection (UTI) managed with nitrofurantoin. She was started on a heparin bridge to warfarin on POD 4. She was discharged home on POD 12.

### Case 5

Patient 5, whose case was previously described by Flaherty et al, 4 was a 46-year-old man with a history of ATAAD status after ascending aorta and aortic arch replacement 2 years prior that was complicated by stroke, atrial fibrillation on apixaban 5 mg twice a day, chronic kidney disease with a baseline creatinine 2.5, and migraines who presented with acute onset left frontal headache and voice hoarseness. He presented 2 days after the onset of symptoms and the last took his apixaban the morning of presentation. CTA demonstrated a 3.5-cm pseudoaneurysm of the aortic root with a surrounding 8 × 8 × 15 cm hematoma. His heparin-calibrated anti-Xa activity level the night of his admission was 1.78 IU/mL (discordant assay, therapeutic range 0.3–0.7 IU/mL). Ten hours preoperatively, he received a 400 mg bolus of andexanet-alfa followed by a continuous infusion at 4 mg/min for 2 hours.

He was taken to the OR for surgical exploration the next morning. Prior to induction, his heparin-calibrated anti-Xa activity level was 1.69 IU/mL, so an additional 400 mg bolus of andexanet-alfa was administered followed by continuous infusion at 4 mg/min for 2 hours, which completed 40 minutes prior to initiation of CPB. Given the proximity of the pseudoaneurysm to the sternum, he was peripherally cannulated and CPB was initiated via the femoral artery and vein prior to sternotomy. Notably, during heparization, 80,000 units of heparin were required to reach an activated clotting time of 434. After sternotomy, a leak was identified at a singular pleat in the noncoronary sinus and was subsequently repaired. After weaning from CPB and reversing with protamine, excellent hemostasis was noted and the sternum was closed without complication. Intra-operatively, the patient received desmopressin but no blood products or factors.

His postoperative course was notable for difficult pain control and dysphagia but was otherwise uncomplicated. He was restarted on apixaban on POD 2, and he was discharged home on POD 8.

## Discussion

Herein, we describe the cases of five patients who were taking DOACs and received urgent or emergent surgery at our institution for ATAAD. Of these patients, four were taking apixaban and one was taking rivaroxaban. This case series is notable for the preoperative use of andexanet-alfa to reverse apixaban in one case. Notably, there was evidence of heparin insensitivity in this case, but there was no intraoperative thrombosis and the patient's postoperative course was not complicated by bleeding or thrombosis. The remainder of cases were successfully managed pharmacologically with administration of procoagulant factors, including 4F-PCC, FFP, and aPCC to replete factor X, as well as fibrinogen.

Review of the literature for case reports describing the use of DOAC reversal agents for surgical repair of ATAAD yielded nine articles describing thirteen separate cases, summarized in Table 2 . 1 2 4 5 6 7 8 9 10 11 12 Of these cases, eight patients were taking direct Xa-inhibitors and received andexanet-alfa, whereas five were taking a direct thrombin inhibitor and received idarucizumab. Of the patients who received andexanet-alfa, six received it either before or on CPB or extracorporeal membrane oxygenation, whereas two received it post-CPB. Of these six patients, all but one had heparin insensitivity or pump thrombosis after administration of andexanet-alfa. The two patients who received andexanet-alfa after CPB did not have any evidence of thrombosis.

**Table 2:** Review of case reports of patients who presented with acute Type A aortic dissection on direct oral anticoagulants and received reversal agents

Age	Extent of dissection	Operation	DOAC	Reversal agent	Others	Timing	Heparin insensitivity or thrombosis	Disposition	References
65	ATAAD	Replacement of ascending aorta, repair of aortic valve, and LAA exclusion	Rivaroxaban 20 mg	Andexanet-alfa 400 mg bolus	Not specified	Post-CPB	No	Alive at 30 d	Al-Attar et al
68	ATAAD	Replacement of ascending aorta	Edoxaban 60 mg	Andexanet-alfa 400 mg bolus	Not specified	Post-CPB	No	Alive at 30 d	Al-Attar et al
75	ATAAD	Replacement of ascending aorta and repair of aortic valve,	Edoxaban 60 mg	Andexanet-alfa 400 mg bolus	No blood products	Pre-CPB	Heparin insensitivity	Alive at 30 d	Al-Attar et al
67	ATAAD with tamponade	Aortic root and hemiarch repair	Apixaban (dose not specified)	Andexanet-alfa 800 mg over 30 minute	3779 u 4F-PCC, entire pump volume exchange with pRBCs and FFP, 500 IU ATIII, extensive additional blood product transfusion	During CPB	Pump thrombosis and heparin insensitivity	Alive at 3 d	Brenner et al
76	ATAAD	Replacement of ascending aorta and hemiarch with descending aortic elephant trunk	Apixaban 2.5 mg daily	Andexanet-alfa 400 mg bolus, 4 mg/min continuous for 66 min	2193 IU 4F-PCC, 4u FFP	Pre-CPB	Heparin insensitivity	Expired POD 11	Brenner et al
81	Unspecified acute aortic syndrome	Replacement of aortic arch	Edoxaban 30 mg daily	Andexanet-alfa 400 mg bolus, 4 mg/min continuous for 2 h	Tranexamic acid, 3000 IU ATIII	Pre-CPB	Heparin insensitivity	Not specified	Honda et al
69	ATAAD with pericardial tamponade and extension to level of iliac arteries	Bentall with ascending and hemiarch replacement	Apixaban 5 mg twice a day	Andexanet-alfa 800 mg over 30 min and 8 mg/min over 2 h	4.1 L cell-saver blood, 13 u pRBC, 17 u FFP, 4 u platelets, 7 g fibrinogen, 3,500 IU PCC, 30 µg desmopressin, 100 mL 10% Ca-gluconate	Post-CPB, on ECMO	Not overt, but had spinal infarction	Alive at time of writing	Kainz et al
46	Aortic root pseudoaneurysm with contrast extravasation	Aortic root repair	Apixaban 5 mg twice a day	Andexanet-alfa, 400 mg bolus, 4 mg/min continuous for 2 h, additional bolus dose	No blood products or factors	Preoperative	Heparin insensitivity, no thrombosis	Alive at discharge	Flaherty et al
79	ATAAD from aortic root to femoral arteries	Replacement of aortic root and bioprosthetic aortic valve, reimplantation of coronary arteries, and placement of aortic endovascular graft	Dabigatran 150 mg twice a day	Idarucizumab 5 g	Not specified	Pre-CPB	No	Intraoperative mortality due to right ventricle failure	Henderson et al
66	ATAAD with aortic insufficiency and pericardial effusion and aortic aneurysm involving the ascending aorta through abdominal aorta with involvement of renal arteries	Replacement ascending aorta and bioprosthetic aortic valve, left coronary artery reimplantation, and right coronary artery repair	Dabigatran (dose NS)	Idarucizumab 5 g	4 u pRBC, 6 u platelets, 3 u FFP	Pre-CPB	No	Discharged alive on POD 20	Tomaszuk-Kazberuk et al
83	Ascending aortic aneurysm c/b TA IMH	Replacement supracoronary ascending aorta and hemiarch	Dabigatran 110 mg twice a day	Idarucizumab 5 g	12 u platelets, 3 u FFP, 3 u pRBC	Post-CPB	No	Alive at 30 d	Mazur et al
76	ATAAD with cardiac tamponade	Replacement supracoronary ascending aorta and hemiarch	Dabigatran 110 mg twice a day	Idarucizumab 5 g	10 u platelets, 3 u FFP, 2 u pRBCs	Post-CPB	No	Immediate postoperative mortality	Mazur et al
67	ATAAD without AI, malperfusion, or hemopericardium	Replacement of ascending aorta and total arch with frozen-elephant trunk	Dabigatran 150 mg twice a day	Idarucizumab 5 g	No blood products or factors	40 h preoperative	No	Discharged alive POD 7	Hamad et al

Abbreviations: ATAAD, acute Type A aortic dissection; CPB, cardiopulmonary bypass; DOAC, direct oral anticoagulants; ECMO, extracorporeal membrane oxygenation; FFP, fresh-frozen plasma; PCC, prothrombin complex concentrate; POD, postoperative day; pRBC, packed red blood cells.

Of the patients who received idarucizumab, one received it 40 hours before surgery, two received it pre-CPB, and two received it post-CPB; there was no evidence of thrombosis in any of these patients, although there were two fatalities. Although a significant reporting bias is expected, there is additional evidence that the administration of andexanet-alfa pre-CPB is associated with heparin insensitivity or pump thrombosis, whereas this association is not evident for the administration of idarucizumab. 1

**Fig. 1:**
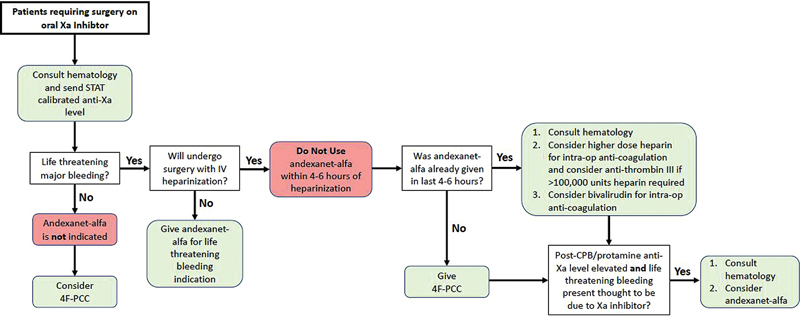
Clinical practice flowchart for management of oral factor Xa inhibitors in patients requiring surgery.

At the authors' institution, patients with normal kidney function are considered to have likely adequately cleared Xa-inhibitors beyond 18 hours after ingestion. Nonetheless, all patients receive a STAT anti-Xa activity level calibrated to the particular anticoagulant ingested and hematology is notified of the case. For patients without major life-threatening bleeding, andexanet-alfa is not indicated, and management can instead proceed with the administration of 4F-PCC. For patients with major life-threatening bleeding and who are expected to undergo surgery requiring intravenous (IV) heparin, andexanet-alfa should not be used within 4 to 6 hours of heparin administration due to its ability to induce heparin insensitivity. Instead, 4F-PCC can be given as an attempt to supply additional factor X. For patients who already received andexanet-alfa but require surgery with IV heparin, hematology is consulted, and clinicians can consider using higher doses of IV unfractionated heparin with possible administration of antithrombin III concentrate if marked heparin insensitivity is observed. They can alternatively consider anticoagulation with bivalirudin. In cases where surgery cannot be delayed, it is standard institutional practice to withhold andexanet-alfa preoperatively but to check anti-Xa activity level again after cessation of CPB and reversal of heparin, and to administer andexanet-alfa if there is life-threatening bleeding and the bleeding is thought to be due to activity of the DOAC ( Fig. 1 ).

For patients who have taken dabigatran and require emergent surgery, idarucizumab 5 g is considered the first-line agent for reversal. When idarcuziumab is not available, 4F-PCC or activated PCC can be administered instead. The description of practice within the authors' institution should not be taken as a fixed protocol nor as a substitute for clinical judgment but does reflect a multidisciplinary review of existing evidence for the management of these agents.

## Conclusion

In our institutional review of patients undergoing surgery for ATAAD with recent ingestion of DOACs, four patients were able to be managed with a combination of factor Xa repletion or surgical delay, while one patient who was treated with andexanet-alfa demonstrated heparin insensitivity. A review of the literature demonstrated a similar safety signal, wherein preoperative andexanet-alfa has been reported to induce heparin insensitivity or thrombosis while on CPB. We recommend against the use of andexanet-alfa in patients who are to undergo surgical aortic repair within 4 to 6 hours but rather recommend treating preoperatively with 4F-PCC or aPCC and consider post-CPB administration of andexanet-alfa if anti-Xa levels are still elevated and there is clinically relevant bleeding.

## Notes

**Erratum:** An erratum has been published for this article (DOI: 10.1055/a-2734-4614)
