# Erratum: Management of Direct Oral Anticoagulants in Acute Type A Aortic Dissection

**DOI:** 10.1055/a-2734-4614

**Published:** 2025-11-14

**Authors:** Robert Semco, Thais Faggion Vinholo, Jake Awtry, Asishana Osho, Kim de la Cruz, Ashraf A. Sabe

**Affiliations:** 1 Harvard Medical School, Boston, Massachusetts; 2 Division of Cardiac Surgery, Department of Surgery, Brigham and Women's Hospital, Boston, Massachusetts; 3 Division of Cardiac Surgery, Department of Surgery, Massachusetts General Hospital, Boston, Massachusetts

It has been brought to the publisher's attention that the name of the author “ *Kim de la Cruz* ” was incorrectly tagged in the HTML version of the above article in the Journal of AORTA, published in Volume 12, Number 6 (DOI: 10.1055/a-2542-4290 ). The tagging has been corrected in the HTML version of the article.

